# Analysis of Gene Signatures of Tumor Microenvironment Yields Insight Into Mechanisms of Resistance to Immunotherapy

**DOI:** 10.3389/fbioe.2020.00348

**Published:** 2020-05-25

**Authors:** Ben Wang, Mengmeng Liu, Zhujie Ran, Xin Li, Jie Li, Yunsheng Ou

**Affiliations:** ^1^Department of Orthopedics, The First Affiliated Hospital of Chongqing Medical University, Chongqing, China; ^2^Graduated School of Anhui University of Traditional Chinese Medicine, Hefei, China; ^3^School of Public Health and Community Medicine, Chongqing Medical University, Chongqing, China; ^4^Department of Respiratory and Critical Care Medicine, The First Affiliated Hospital of Chongqing Medical University, Chongqing, China; ^5^Department of Oncology, The First Affiliated Hospital of Chongqing Medical University, Chongqing, China

**Keywords:** immunotherapy, tumor microenvironment, immunotherapeutic resistance, molecular targeted agents, personalized medicine

## Abstract

**Background:** The recent clinical success of immunotherapy represents a turning point in cancer management. But the response rate of immunotherapy is still limited. The inflamed tumor microenvironment has been reported to correlate with response in tumor patients. However, due to the lack of appropriate experimental methods, the reason why the immunotherapeutic resistance still existed on the inflamed tumor microenvironment remains unclear.

**Materials and Methods:** Here, based on single-cell RNA sequencing, we classified the tumor microenvironment into inflamed immunotherapeutic responsive and inflamed non-responsive. Then, phenotype-specific genes were identified to show mechanistic differences between distant microenvironment phenotypes. Finally, we screened for some potential drugs that can convert an unfavorable microenvironment phenotype to a favorable one to aid current immunotherapy.

**Results:** Multiple signaling pathways were phenotypes-specific dysregulated. Compared to non-inflamed microenvironment, the expression of interleukin signaling pathways-associated genes was upregulated in inflamed microenvironment. Compared to inflamed responsive microenvironment, the PPAR signaling pathway-related genes and multiple epigenetic pathways-related genes were, respectively, suppressed and upregulated in the inflamed non-responsive microenvironment, suggesting a potential mechanism of immunotherapeutic resistance. Interestingly, some of the identified phenotype-specific gene signatures have shown their potential to enhance the efficacy of current immunotherapy.

**Conclusion:** These results may contribute to the mechanistic understanding of immunotherapeutic resistance and guide rational therapeutic combinations of distant targeted chemotherapy agents with immunotherapy.

## Introduction

Although immunotherapy has revolutionized tumor treatment, it still has some limitations (Larkin et al., 2015). For example, the success of adoptive cell therapy (ACT) on hematological malignancies cannot be reproduced on solid tumors (Newick et al., 2017). The responsive rate of immune checkpoint inhibitors (CPIs) varies by tumor type, from 45% for melanoma (Daud et al., 2016; Ribas et al., 2016) to only 12.2% for head neck squamous cancer (HNSC) (Abril-Rodriguez and Ribas, 2017; Darvin et al., 2018).

To better understand the reasons for these limitations, a number of studies tried to investigate the effect of tumor microenvironment (TME) phenotype on immunotherapy and suggested that TME phenotype (broadly categorized as being inflamed or non-inflamed) (Binnewies et al., 2018; Galon and Bruni, 2019) was a critical factor responsible for these limitations (Ji et al., 2012; Peng et al., 2015; Spranger et al., 2015; Chen et al., 2016; Kortlever et al., 2017). However, for the lack of appropriate experimental methods, a systematic understanding of how inflamed TME forms and why therapeutic resistance still exists on inflamed TME has been constrained. Here, to better understand the role of TME phenotypes to aid current immunotherapy, we systematically analyzed pan-cancer molecular characteristics of inflamed TME and further delved into the mechanistic differences between inflamed responsive TME and inflamed non-responsive TME. Importantly, part of our results has been supported in recent reports (Chowdhury et al., 2018; Wang J. et al., 2019).

Together, these results have profound prospects in clinical application, including identifying multiple potential immunotherapeutic targets, providing mechanistic insights into immunotherapeutic resistance in inflamed TME, and screening for some potential immunophenotypic regulation drugs to guide rational combination of chemotherapy agents with immunotherapy.

## Methods

### Pan-Cancer Samples and Clinical Cohorts Treated by Immunotherapy

RNA sequencing data across 19 The Cancer Genome Atlas (TCGA) tumor types were downloaded from the Gene Expression Omnibus (GEO) database with accession number GSE62944 (Rahman et al., 2015). The updated clinical data were downloaded from *TCGAbiolinks* (Colaprico et al., 2016; Silva et al., 2016; Mounir et al., 2019). Published RNA sequencing data (Riaz et al., 2017) of 101 clinical tumor samples treated by anti-CTLA4 and anti-PD1 were downloaded from the GEO database with accession number GSE91061. The raw count data of RNA sequencing were normalized and quantitated by the edgeR package (Robinson et al., 2010).

### Identifying Immune Cell Signature From Integrated Single-Cell RNA Sequencing Data

In order to analyze the TME of different tumor types and increase the diversity of non-immune cell to obtain robust immune cell markers, we applied the Seurat integration pipeline (Butler et al., 2018) to integrate two single-cell RNA sequencing data sets, respectively, from the Puram's HNSC cohort (GEO accession number: GSE103322) (Puram et al., 2017) and Tirosh's melanoma cohort (GSE72056) (Tirosh et al., 2016). A CCA algorithm (Butler et al., 2018) derived from machine learning was used to identify anchors of cells from different tumor types for the purpose of unbiased single-cell data integration (Stuart et al., 2019). Annotations of immune cells referred to the original literature and cell marker database (Tirosh et al., 2016; Puram et al., 2017; Zhang et al., 2019). Immune cell gene signatures (GSs) were defined based on the following criteria: (1) the proportion of signature expression in immune cells (CD8 T cell, CD4 T cell, B cells, macrophage, mast cell, dendritic cell, NK cell) should be >0.6; (2) the percent of GS expression in non-immune cells (myocytes, tumor cells, endothelial, fibroblast) should be <0.3; (3) adjusted *P* < 0.001; (4) log (fold change)>0 (compared to non-immune cells and other immune cell clusters).

### Unsupervised Clustering Algorithm to Determine TME Subtypes of Tumor Samples

Immune cell markers identified in single-cell RNA sequencing analysis were used as an input for the gene set variation analysis (GSVA) algorithm (Hänzelmann et al., 2013) to calculate the immune score for each immune cell. Then, tumor samples were classified into high-immune score (inflamed), intermediate immune score, and low-immune score (non-inflamed) based on the unsupervised clustering pattern. This method has been proven as an efficient way to indirectly evaluate the phenotypes of TME (Wang et al., 2018). By using optCluster (Sekula et al., 2017) to evaluate the internal and stability indexes of the seven clustering algorithms (clara, diana, hierarchical, kmeans, model, pam, and sota), the optimal number and the algorithm of clustering were determined. Finally, the Clara algorithm and three groups were selected as the most robust clustering parameters. To avoid the unfavorable bias of confounding factors, we excluded intermediate immune score samples in further analysis.

### Identification of Altered Signaling Pathways

Differentially expressed genes (DEGs) were identified by edgeR package (Robinson et al., 2010) with a negative binomial distribution algorithm; *P* < *0.05* and an absolute value of log2-fold change >1.5 were considered as statistically significant. Then, we annotated these DEGs with ClusterProfile (Yu et al., 2012) and RectomePA (Yu and He, 2016) package according to KEGG and Rectome pathway databases. Gene set enrichment analysis (GSEA) was used to provide a systematic view into molecular pathway alternation (Subramanian et al., 2005). ToPASeq package was used to provide topology-based pathway analysis (Ihnatova and Budinska, 2015).

### Screening for Potential Phenotype Transformation Drugs

To discover potential drugs aiding current immunotherapy, we calculated the connectivity score (Lamb et al., 2006) of multiple drugs to evaluate whether it is promising to promote the transformation of favorable TME phenotypes. This analysis was carried on *PharmacoGx* packages (Smirnov et al., 2015).

### Statistical Analysis

To assess the prognostic significance of TME subtypes, we used a Cox test to calculate its hazard ratio. Then, Kaplan–Meier curves and log-rank test were used to assess the differences in the 5 years' and all years' overall survival times between inflamed and non-inflamed subtypes. Pearson's chi-square test and Fisher's exact test were used to calculate the *P*-value for the discrete variable. A *P* < 0.05 was regarded as statistically significant.

## Results

### Integration of Single-Cell RNA Sequencing Data Sets

**Figure 1:**
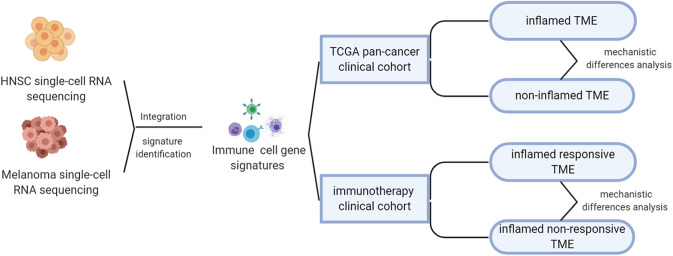
Overall design of this study.

The overall design of this study was shown in Figure 1. As mentioned above, the responsive rate of immunotherapy varies by tumor type. To understand the factors that contribute to the differences in susceptibility to immunotherapy, we integrated two single-cell RNA sequencing datasets, respectively, from head and neck squamous carcinoma (HNSC) and melanoma, which were characterized by different immunotherapeutic sensitivity (~45% response rate for melanoma Daud et al., 2016; Ribas et al., 2016, significantly higher than the 12.2% of HNSC Wang B. C. et al., 2019).

**Figure 2:**
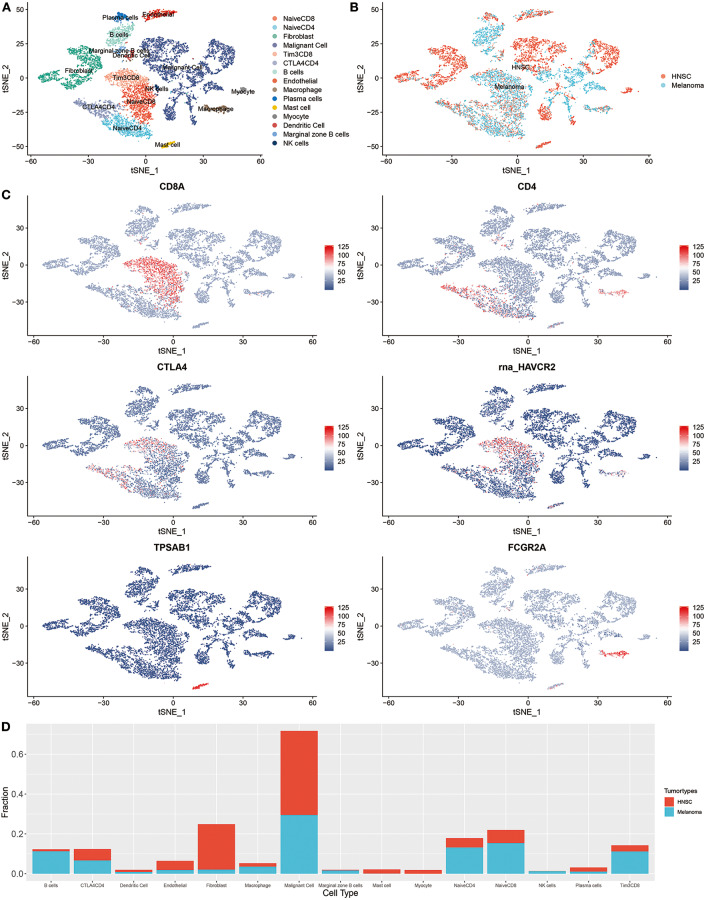
Integrated single-cell RNA sequencing analysis revealed the microenvironment heterogeneity of distant tumor types. **(A)** The t-SNE plot displays immunological and non-immunological cells in the tumor microenvironment. Each dot represents a cell and color represents different types of cells. **(B)** The color was coded according to tumor types. **(C)** The expression of cell markers across different cell clusters. **(D)** The composition of cells in HNSC and melanoma.

The integration result is shown in Figures 2A–C; tumor cells from HNSC and melanoma exhibited significant heterogeneity. Nevertheless, immune cells from different tumor types were integrated into corresponding immune cell clusters. These results suggested that immune cells from distant tumor types might have a relatively similar transcriptomic pattern, which may explain the reason why immunotherapy was always accompanied by a pan-cancer therapeutic effect. The heterogeneity of immunotherapeutic efficacy across distant tumor types may be mainly derived from different tumor cells and their tumor immune microenvironment characteristics, such as immune cell composition.

For instance, B cells are increasingly valued for their important role in immunotherapeutic resistance (Petitprez et al., 2020). As shown in Figure 2D, the proportion of B cells in melanoma was significantly higher than that of HNSC (*P* < 0.001, Supplementary Table 1).

### Pan-Cancer Prognostic Significance of TME Subtypes

**Figure 3:**
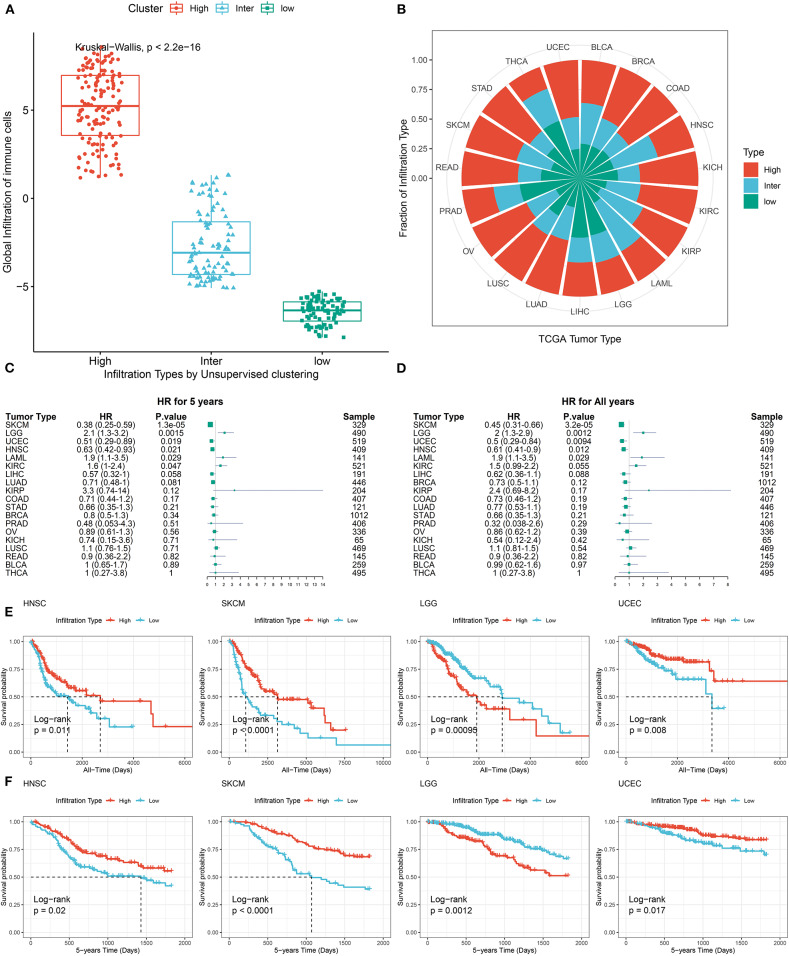
Pan-cancer prognostic role of identified TME subtypes. **(A)** The global infiltration characteristics of distant TME subtypes. **(B)** The proportion of TME subtypes in different cancer types. **(C,D)** Forest plot for the association between identified TME subtypes and overall survival time of patients. **(E,F)** All years or 5 years overall survival time of inflamed (High) and non-inflamed (Low) TME. Tumor types were represented by TCGA. Standard abbreviations: LAML, acute myeloid leukemia; BLCA, bladder urothelial carcinoma; LGG, brain lower grade glioma; BRCA, breast invasive carcinoma; COAD, colon adenocarcinoma; KICH, kidney chromophobe; KIRC, kidney renal clear cell carcinoma; KIRP, kidney renal papillary cell carcinoma; LIHC, liver hepatocellular carcinoma; LUAD, lung adenocarcinoma; LUSC, lung squamous cell carcinoma; OV, ovarian serous cystadenocarcinoma; PRAD, prostate adenocarcinoma; READ, rectum adenocarcinoma; SKCM, skin cutaneous melanoma; STAD, stomach adenocarcinoma; THCA, thyroid carcinoma; UCEC, uterine corpus endometrial carcinoma.

To classify TME phenotypes across distant tumor types, immune cell GSs were identified in the above single-cell data. Then, we classified TCGA pan-cancer samples into three TME subtypes based on the unsupervised clustering pattern of GS, each assigned as high-immune score (inflamed), intermediate immune score, or low-immune score (non-inflamed; Figure 3A). As shown in Figure 3B, the proportions of TME subtypes varied greatly among the different types of tumors. Next, we examined the association of this classification with the overall survival time of tumor patients. Consistent with previous reports from immunohistochemistry (Dubsky et al., 2019), favorable prognostic roles of inflamed TME were observed in most tumor types (such as SKCM, UCEC, etc.). Unexpectedly, as reported in a number of previous reports, an unfavorable prognostic role of inflamed TME was also observed in some tumor types, such as LGG (Zhang et al., 2017) (Figures 3C–F).

### Molecular Characteristics of Inflamed or Non-inflamed TME Across Multiple Tumor Types

**Figure 4:**
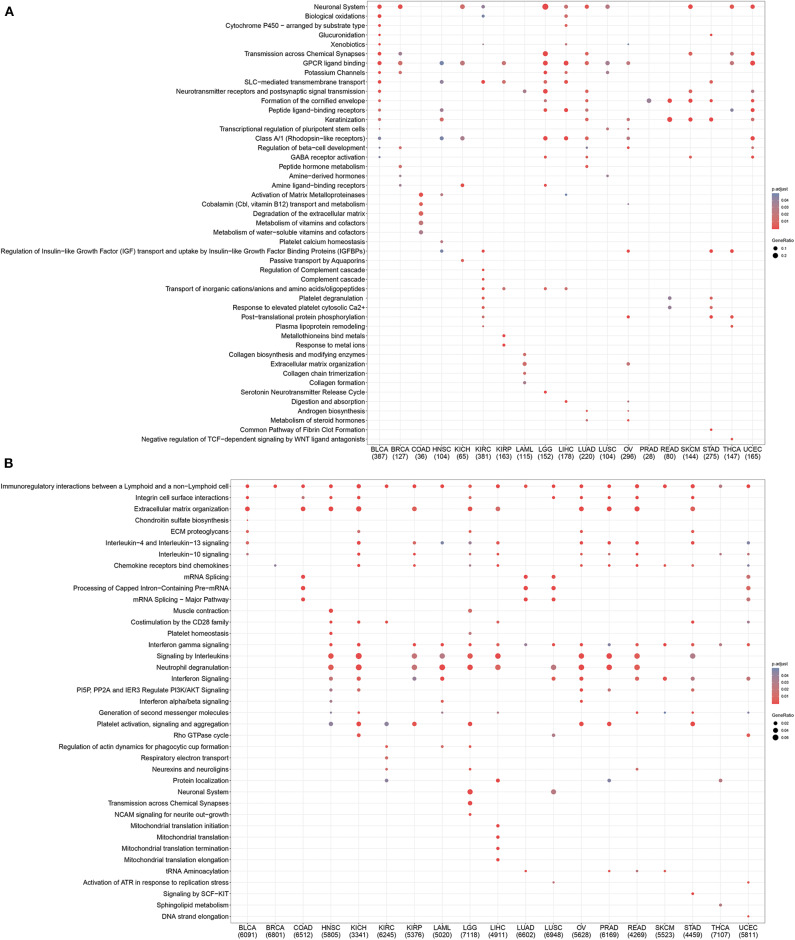
Pan-cancer gene functional annotation of TME phenotype associated genes. **(A)** Gene function of upregulated genes in non-inflamed TME. **(B)** The function of inflamed TME associated genes. The number under abbreviation represents the number of differently expressed genes (DEGs).

To further investigate mechanistic differences between inflamed and non-inflamed TME, we compared gene expression profiles between inflamed and non-inflamed TME. As shown in Figure 4A, non-inflamed TME-specific genes (upregulated genes in non-inflamed TME) were related to the GPCR signaling pathway, neuronal system, and keratinization. Inflamed TME-specific genes (upregulated genes in inflamed TME) were related to interferon (IFN), multiple interleukin-related pathways including interleukin-4, interleukin-13, and interleukin-10 signaling, CD28 costimulatory molecule family including PD-1, and CTLA-4-associated signaling pathways (Figure 4B). The topology-based pathway analysis demonstrated that interleukin-related pathways, interferon-related pathways, the NLRP3 inflammasome, Toll-like receptor, mitochondria, CD28 costimulation, and B cell activation-related pathways were also activated in inflamed TME (Supplementary Table 5).

### TME Phenotypes Correlated With the Immunotherapeutic Sensitivity

**Figure 5:**
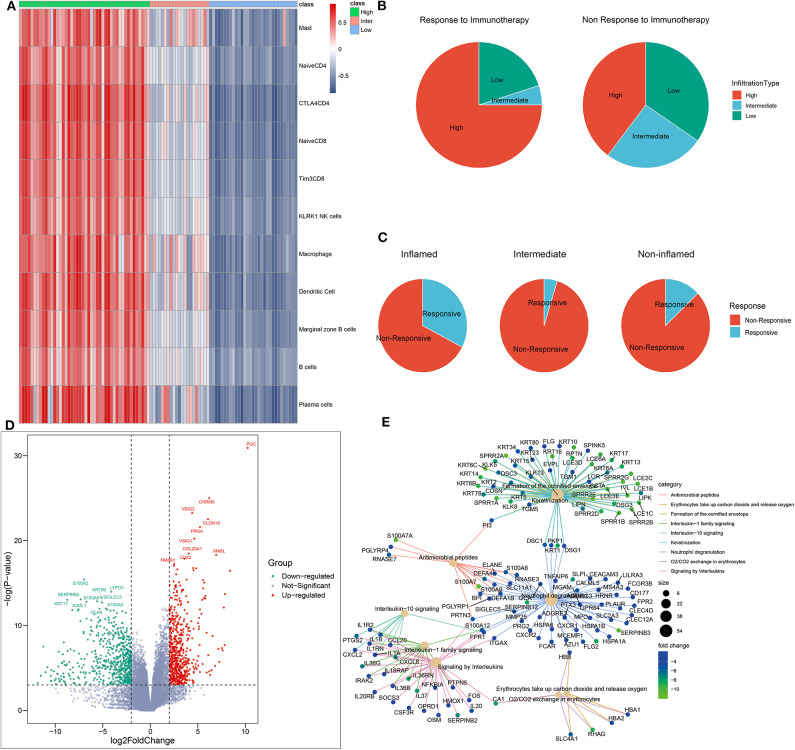
Identification of phenotype-specific genes. **(A)** Heatmap of distant TME subtypes determined by unsupervised clustering algorithm. **(B,C)** TME subtypes correlated with the response to immunotherapy. **(D)** The volcano plot of differently expressed genes based on an RNA sequencing analysis of inflamed responders vs. inflamed non-responders. Red and green dots, respectively, represented upregulated genes and downregulated genes in inflamed responders. **(E)** The network plot showing common genes shared by top functional terms of upregulated genes in inflamed non-responders.

To better understand the association between TME phenotypes and the response to immunotherapy, we reproduced our TME classification in a published clinical melanoma cohort treated by immune CPIs (Riaz et al., 2017) (Figure 5A). This reproduction was performed based on immune GSs identified in the above single-cell RNA sequencing analysis with the same clustering parameter.

As expected, inflamed tumors were the most sensitive to CPI (CR+PR rate: 32.6% in inflamed vs. 3.2% in non-inflamed, *P* = 0.015, Supplementary Table 2) (Figure 5B), but only a percentage (CR rate: 8.7%, CR + PR rate: 32.6%) of these patients were responsive to CPI (Figure 5C).

To further offer mechanistic insights into CPI resistance in inflamed TME, we identified several DEGs in inflamed non-responders vs. inflamed responders (Figure 5D). These GSs of TME phenotype may serve as potential targets for improving current immunotherapy.

For instance, CLDN18 was the signature of inflamed responsive TME. Therapy that directly targets on CLDN18 has shown its potential to improve the efficacy of ACT in treating solid tumors (Micke et al., 2014). On the other side, inhibiting the signature of inflamed non-responsive TME may be another promising way. Here, SIGLEC5 was significantly overexpressed in inflamed non-responders, and its family member SIGLEC15 has been proven as an efficient target to enhance antitumor immunity (Wang J. et al., 2019).

We also analyzed the correlation between TME status and known ICB response biomarkers. The inflamed TME was characterized by higher expression of PDCD1, CTLA4, CD28, (PD-L1) CD274, PD-L2, and lower tumor mutation burden than non-inflamed TME (Supplementary Figures 3, 4).

### Mechanistic Differences Between Inflamed Responsive TME and Inflamed Non-responsive TME

**Figure 6:**
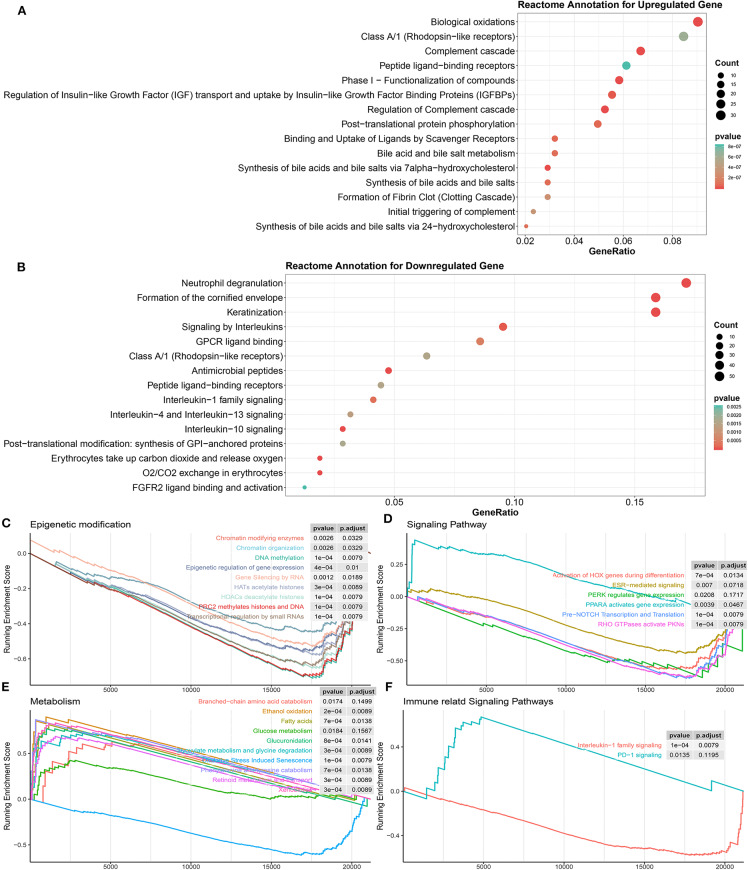
Biological processes correlated with identified DEGs in inflamed responders. **(A)** Detailed function annotation of upregulated genes in inflamed responders (compared to inflamed non-responders). **(B)** Functional annotation of downregulated genes in inflamed responders (compared to inflamed non-responders). **(C–F)** GSEA shows four dimensions of the molecular function of DEGs across inflamed responders. Running enrichment score >0 means this pathway is upregulated in inflamed responders; running enrichment score <0 means this pathway is downregulated in inflamed responders.

Then, gene functional annotation analysis was used to understand the role of TME phenotype-specific genes. As shown in Figure 6A, genes upregulated in inflamed and responsive tumors enriched on complement cascade and bile metabolism. GSEA also confirmed that multiple metabolism associated pathways except for oxidative stress induced senescence were upregulated in this type of TME, including bile salt and bile acid metabolism, glucose metabolism, ethanol oxidation, glyoxylate metabolism, and glycine degradation (Figure 6E).

In terms of inflamed non-responsive tumors, signaling pathways, such as IL-13, IL-4, IL-10, and IL-1 cytokines-related signaling pathways and oxygen exchange pathway were upregulated, which are also downregulated in inflamed responders (Figures 5E, 6B). Interestingly, the expression of CTLA-4 pathway-related genes did not differ between inflamed responders and inflamed non-responders (Supplementary Table 4).

The topology-based pathway analysis demonstrated that the B cell activation pathway, non-canonical NF-kB pathway, NOTCH signaling pathways, PD-1 signaling, bile acid, and bile salt metabolism-related pathways were inhibited in inflamed non-responders (Supplementary Table 6).

These results suggested that tumor hypermetabolism might confer resistance to immunotherapy.

Finally, for a more systematic understanding of the resistant mechanism, we applied GSEA to investigate the alternation of molecular pathways across four dimensions (epigenetic modification, immune or other associated signaling pathway, metabolism).

As shown in Figure 6C, multiple epigenetic signaling pathways were upregulated in inflamed non-responders, which suggested a mechanism of immunotherapeutic resistance as observed by others (Mondello et al., 2020; Olino et al., 2020).

In terms of inflamed responders, multiple carcinogenesis signaling pathways, except for the PPAR pathway, were downregulated (Figures 6D,F), which suggested a mechanism of therapeutic resistance and potential target for therapy. In line with this hypothesis, recent studies illustrated that PPAR agonists appeared to improve the therapeutic sensitivity of ACT and CPI therapy (Chowdhury et al., 2018; Saibil et al., 2019).

A deeper analysis of differentiating the patient population between different ICB treatments demonstrated that 135/980 (13.78%) pathways enriched on the CTLA-4 cohort were also enriched on the PDCD1 cohort (135/673, 20.06%) (Supplementary Figure 1). The shared pathways enriched on two cohorts were associated with glucuronidation, interleukin-10 signaling, O2/CO2 exchange in erythrocytes, post-translational phosphorylation, and metabolism of bile acids and bile salts (Supplementary Figure 2A). Genes dysregulated in the CTLA-4 cohort tended to be associated with epigenetic modification including epigenetic regulation of gene expression, HATs acetylate histones, HDAC deacetylates histones, transcriptional regulation by small RNAs, and gene silencing by RNA (Supplementary Figure 2B). Genes dysregulated in the PDCD1 cohort tended to be associated with PPAR active gene expression, glucose metabolism, extracellular matrix organization, GPCR ligand binding, and signaling by retinoic acid (Supplementary Figure 2C).

### Screening for Potential Favorable TME Phenotype Transformation Drugs

Immunotherapy combined with chemotherapy is receiving increasing interest as a promising strategy to improve the deficiencies of current immunotherapy (Wargo et al., 2015). However, it is not completely clear how best to incorporate chemotherapy with immunotherapy. Here, we calculated the genomic connectivity score of 1,288 kinds of drugs to identify potential phenotype transformation drugs that could induce systemic favorable transcriptomic alternation, including from non-inflamed TME to inflamed TME, or from inflamed non-responsive TME to inflamed responsive TME. The drug-genomic perturbation database records genomic changes following multiple drug treatments. Analysis combining these drug-induced genomic changes with identified phenotypic genomic differences can help us find potential drugs that could convert unfavorable TME to favorable TME.

Mercaptopurine (6-MP) was identified as the most promising drug that might promote the transformation of inflamed responsive TME phenotype (Table 1). Interestingly, although some reports have shown that 6-MP can enhance the vaccine-dependent antitumor immunit y (Kataoka et al., 1984; Kataoka and Oh-hashi, 1985), it seems to be forgotten after that. But there are increasing interests trying to use 6-MP as a drug of immune disorders, such as autoimmune hepatitis (Hübener et al., 2016), inflammatory bowel disease (Present et al., 1989), etc. This may be because 6-mercaptopurine is widely recognized as an immunosuppressive agent, but our findings implicated that immunomodulatory may be a more accurate definition of such drugs. Our results indicated that further clinical studies are needed to assess the value of the combination of 6-MP with current immunotherapy.

**Table 1:** Screening for potential favorable TME phenotype transformation drugs.

**Potential drugs that convert “non-inflamed” TME to “inflamed” TME**			**Potential drugs that convert “inflamed nonresponsive” TME to “inflamed responsive” TME**		
**Drugs**	**Connectivity**	***P-*Value**	**Drugs**	**Connectivity**	***P-*Value**
Clofibrate	0.803	0.021	Mercaptopurine	0.817	0.028
Metronidazole	0.637	0.002	Valdecoxib	0.309	0.040
Zalcitabine	0.605	0.028	5253409	0.292	0.006
Gabexate	0.585	0.012	Astemizole	0.290	0.039
S-propranolol	0.581	0.007	Isoconazole	0.283	0.003
Ifenprodil	0.580	0.044	Pizotifen	0.279	0.011
Sulfapyridine	0.578	0.027	Econazole	0.278	0.003
Succinylsulfathiazole	0.570	0.042	Orciprenaline	0.267	0.006

## Discussion

Molecular stratification of TME phenotypes is paving the way for a better understanding of immunotherapeutic heterogeneity. Here, based on immune GSs developed from integrated single-cell RNA sequencing analysis, we systematically analyzed the molecular characteristics of inflamed TME across multiple cancer types and provided mechanistic insights into immunotherapeutic resistance in inflamed TME.

Some of the identified mechanistic differences have been supported by recent reports. Examples highlighted by these data include the upregulation of epigenetic signaling pathways and the downregulation of PPAR-signaling pathways in inflamed non-responsive tumors. These dysregulated pathways may be potential targets for improving the sensitivity to immunotherapy. Importantly, these results are in line with prior publications, which have provided some evidence that inhibition of epigenetic modification (Mondello et al., 2020) or activation of PPAR signaling pathways (Chowdhury et al., 2018; Saibil et al., 2019) might be a promising way to overcome therapeutic resistance to immune checkpoint blockade or ACT.

Our results also revealed the molecular characteristics of inflamed TME shared by different tumor types. These results demonstrated that inflamed TME was related to enhanced cytokine expression (interferon and IL-4,−13, and−10). Interestingly, these cytokines, except for interferon, were also upregulated in inflamed non-responders, which suggested a dual role of these interleukins. These results are in line with prior published reports (Mannino et al., 2015; Wang et al., 2016). For example, IL-10 is widely recognized as an immunosuppressive cytokine, but there is increasing evidence that it has a dual role in antitumor immunity. Blocking or activation of IL-10 has been proven as an efficient way to enhance antitumor immunity in different aspects (Ni et al., 2015; Naing et al., 2018). According to our results, we believe that TME phenotypes should be considered as a key factor in further study design to illuminate the remaining mysteries of IL-10.

In addition, our results have far-reaching clinical implications including the identification of multiple potential molecular targets for developing novel immunotherapy and combination therapeutic strategies. For instance, the success of ACT cannot be reproduced on solid tumors due to the obstacle of its microenvironment. Therefore, rather than directly targeting on whole solid tumors, selectively targeting the inflamed and responsive TME might be another easier therapeutic way. As expected, this hypothesis is supported by a recent report. CLDN18, a signature of inflamed and responsive TME, has been proven as an efficient target for improving the efficacy of current ACT on solid tumors (Micke et al., 2014).

Except for targeting on inflamed and responsive TME, examples highlighted by our data also included inhibiting the signature of inflamed non-responsive TME to reverse therapeutic resistance. For example, SIGLEC15, a signature of inflamed and non-responsive TME, has shown its power in blocking immune escape. Interestingly, its antitumor immunity enhancement effect is independent of the PD-1/PD-L1 axis, suggesting that it may be an ideal target to aid current anti-PD-1 therapy (Wang J. et al., 2019).

Finally, based on a drug-genomic perturbation database, we identified some drugs that were promising for promoting the transformation from an unfavorable TME phenotype to a favorable one.

In conclusion, our result provided an important view for understanding how inflamed TME and inflamed resistant TME form. This evidence has important clinical implications and may help guide rational combination immunotherapy.

## Data Availability Statement

Publicly available datasets were analyzed in this study. This data can be found here: GEO:https://www.ncbi.nlm.nih.gov/gds/.

## Author Contributions

BW and YO designed and supervised the study and was a major contributor in editing the manuscript. BW, ZR, and ML analyzed and interpreted the data and were major contributors in writing the manuscript. BW, XL, and JL performed analysis and contributed to writing the manuscript. All authors read and approved the final manuscript.

## Conflict of Interest

The authors declare that the research was conducted in the absence of any commercial or financial relationships that could be construed as a potential conflict of interest.
