# Supplementary material for: Analysis of Gene Signatures of Tumor Microenvironment Yields Insight Into Mechanisms of Resistance to Immunotherapy
Source: Front Bioeng Biotechnol. 2020 May 25;8:348. doi: 10.3389/fbioe.2020.00348 (PMC7263059; doi:10.3389/fbioe.2020.00348)
Supplement: Supplementary file 1 [file Data_Sheet_1.docx]

***Supplementary material***

**1 Supplementary Figures and Tables**

**1.1. Supplementary Tables**

**Supplementary Table 1. The composition of tumor microenvironment in HNSC and SKCM single cell RNA sequencing data.**

| **Cell types** | **HNSC(n=5902)** | **SKCM(n=4645)** | ***X*** | ***P.value*** |
| --- | --- | --- | --- | --- |
| B cells | 59 | 527 | 528.2396893 | 6.82E-117 |
| CTLA4 CD4 T cells | 334 | 309 | 4.307170958 | 0.037952026 |
| Dendritic cells | 67 | 43 | 0.911540313 | 0.339706058 |
| Endothelial cells | 271 | 89 | 55.63504576 | 8.73E-14 |
| Fibroblast | 1347 | 99 | 938.9238292 | 3.39E-206 |
| Macrophage | 99 | 168 | 38.84040797 | 4.60E-10 |
| Malignant Cell | 2493 | 1371 | 180.7422666 | 3.34E-41 |
| Marginal zone B cells | 17 | 80 | 57.11414748 | 4.11E-14 |

**Supplementary Table 2. Mutation status and responsive rate of different infiltration subtypes.**

| **Characteristics** | **High(n=35)** | **Intermediate(n=21)** | **Low(n=31)** | ***P.value*** |
| --- | --- | --- | --- | --- |
| **Mutation status** |  |  |  |  |
| BRAF | 18(0.419) | 4(0.211) | 4(0.148) | 0.036 |
| NF1 | 1(0.023) | 1(0.053) | 3(0.111) | 0.298 |
| RAS | 9(0.209) | 2(0.105) | 6(0.222) | 0.558 |
| Triple Wt | 15(0.349) | 12(0.632) | 14(0.519) | 0.092 |
| **Response** |  |  |  |  |
| CR Rate | 4(0.087) | 1(0.048) | 1(0.032) | 0.592 |
| CR+PR Rate | 15(0.326) | 1(0.048) | 4(0.129) | 0.015 |

**Supplementary Table 3. Report of optCluster algorithm.**

| Clustering method | Validation Measures: |  |  |  |  |  |
| --- | --- | --- | --- | --- | --- | --- |
|  | Number of clusters | 2 | 3 | 4 | 5 | 6 |
| clara | APN | 0.0343 | 0.0018 | 0.0407 | 0.0322 | 0.0364 |
|  | AD | 0.9959 | 0.7239 | 0.6037 | 0.5569 | 0.5232 |
|  | ADM | 0.1065 | 0.0029 | 0.0555 | 0.0376 | 0.0373 |
|  | FOM | 0.2490 | 0.1780 | 0.1461 | 0.1404 | 0.1305 |
|  | Connectivity | 4.0659 | 13.2008 | 16.5575 | 28.1333 | 33.7548 |
|  | Dunn | 0.2516 | 0.2384 | 0.1254 | 0.0786 | 0.0934 |
|  | Silhouette | 0.6370 | 0.5659 | 0.5091 | 0.4432 | 0.4287 |
| diana | APN | 0.0068 | 0.1350 | 0.0116 | 0.0369 | 0.0597 |
|  | AD | 0.9644 | 0.8892 | 0.5998 | 0.5798 | 0.5595 |
|  | ADM | 0.0214 | 0.3517 | 0.0168 | 0.0519 | 0.0691 |
|  | FOM | 0.2421 | 0.2002 | 0.1472 | 0.1475 | 0.1447 |
|  | Connectivity | 4.0659 | 13.2496 | 16.3206 | 22.1663 | 35.6258 |
|  | Dunn | 0.2516 | 0.1253 | 0.1782 | 0.1831 | 0.1509 |
|  | Silhouette | 0.6370 | 0.4900 | 0.5087 | 0.5091 | 0.4572 |
| hierarchical | APN | 0.1254 | 0.1156 | 0.0513 | 0.1227 | 0.1486 |
|  | AD | 1.2101 | 0.8463 | 0.6133 | 0.5904 | 0.5657 |
|  | ADM | 0.4093 | 0.2920 | 0.0677 | 0.1328 | 0.1736 |
|  | FOM | 0.2788 | 0.1989 | 0.1497 | 0.1437 | 0.1408 |
|  | Connectivity | 3.0710 | 9.9940 | 14.0599 | 23.0413 | 32.1623 |
|  | Dunn | 0.1395 | 0.0857 | 0.1254 | 0.1254 | 0.1158 |
|  | Silhouette | 0.5598 | 0.4758 | 0.5038 | 0.4454 | 0.3243 |
| kmeans | APN | 0.0091 | 0.0582 | 0.0163 | 0.1274 | 0.1658 |
|  | AD | 0.9663 | 0.7552 | 0.5909 | 0.5895 | 0.5676 |
|  | ADM | 0.0279 | 0.1213 | 0.0200 | 0.1426 | 0.1916 |
|  | FOM | 0.2423 | 0.1840 | 0.1433 | 0.1409 | 0.1397 |
|  | Connectivity | 4.0659 | 19.3603 | 16.5575 | 25.3607 | 34.8123 |
|  | Dunn | 0.2516 | 0.1489 | 0.1254 | 0.1271 | 0.0807 |
|  | Silhouette | 0.6370 | 0.5441 | 0.5091 | 0.4514 | 0.3371 |
| model | APN | 0.0248 | 0.0747 | 0.1161 | 0.0445 | 0.1451 |
|  | AD | 1.1108 | 0.8301 | 0.7567 | 0.6473 | 0.6418 |
|  | ADM | 0.0637 | 0.1380 | 0.1834 | 0.0771 | 0.1501 |
|  | FOM | 0.2945 | 0.2124 | 0.1903 | 0.1767 | 0.1692 |
|  | Connectivity | 18.1397 | 43.1567 | 44.5631 | 61.4433 | 69.7917 |
|  | Dunn | 0.0664 | 0.0638 | 0.0485 | 0.0519 | 0.0519 |
|  | Silhouette | 0.5578 | 0.4523 | 0.3328 | 0.3138 | 0.2222 |
| pam | APN | 0.0508 | 0.0107 | 0.0279 | 0.0433 | 0.1120 |
|  | AD | 1.0158 | 0.7432 | 0.5977 | 0.5610 | 0.5444 |
|  | ADM | 0.1588 | 0.0219 | 0.0356 | 0.0486 | 0.1012 |
|  | FOM | 0.2528 | 0.1882 | 0.1449 | 0.1419 | 0.1361 |
|  | Connectivity | 4.0659 | 20.0738 | 18.3782 | 28.1333 | 33.7548 |
|  | Dunn | 0.2516 | 0.1426 | 0.0941 | 0.0786 | 0.0934 |
|  | Silhouette | 0.6370 | 0.5246 | 0.5037 | 0.4432 | 0.4287 |
| sota | APN | 0.0073 | 0.0073 | 0.0173 | 0.0223 | 0.0510 |
|  | AD | 0.9643 | 0.7587 | 0.5968 | 0.5642 | 0.5446 |
|  | ADM | 0.0223 | 0.0162 | 0.0231 | 0.0272 | 0.0623 |
|  | FOM | 0.2417 | 0.1918 | 0.1469 | 0.1411 | 0.1368 |
|  | Connectivity | 4.0659 | 7.1369 | 16.3194 | 26.3087 | 37.5488 |
|  | Dunn | 0.2516 | 0.1933 | 0.1438 | 0.1477 | 0.1847 |
|  | Silhouette | 0.6370 | 0.5483 | 0.5077 | 0.4853 | 0.4403 |

The overall optimal clustering method and number of clusters is:

clara-3

The optimal list is:

clara-3 diana-2 diana-4 sota-2 kmeans-2 sota-3 pam-4 sota-4 sota-5 kmeans-4

diana-5 sota-6 clara-4 pam-3 clara-5 diana-6 clara-6 pam-5 pam-2 kmeans-3 clara-2

hierarchical-4 pam-6 kmeans-5 hierarchical-5 hierarchical-6 model-2

diana-3 hierarchical-2 model-5 model-3 kmeans-6 model-4 model-6

Algorithm: CE

Distance: Spearman

Score: 76.23718

Iterations: 331

**Supplementary Table 4. GSEA analysis for CTLA4 inhibitory signaling pathway.**

| ID | Description | NES | pvalue | p.adjust | qvalues | rank |
| --- | --- | --- | --- | --- | --- | --- |
| R-HSA-389513 | CTLA4 inhibitory signaling | -0.4889913 | 0.9915702 | 1 | 0.8966583 | 1269 |

**Supplementary Table 5. The topology-based pathway analysis for pan-cancer cohort.**

This table can be accessed in https://figshare.com/articles/Supplementary_table/12027138

**Supplementary Table 6. The topology-based pathway analysis for immunotherapy cohort.**

This table can be accessed in https://figshare.com/articles/Supplementary_table/12027138

**1.2. Supplementary Figures**


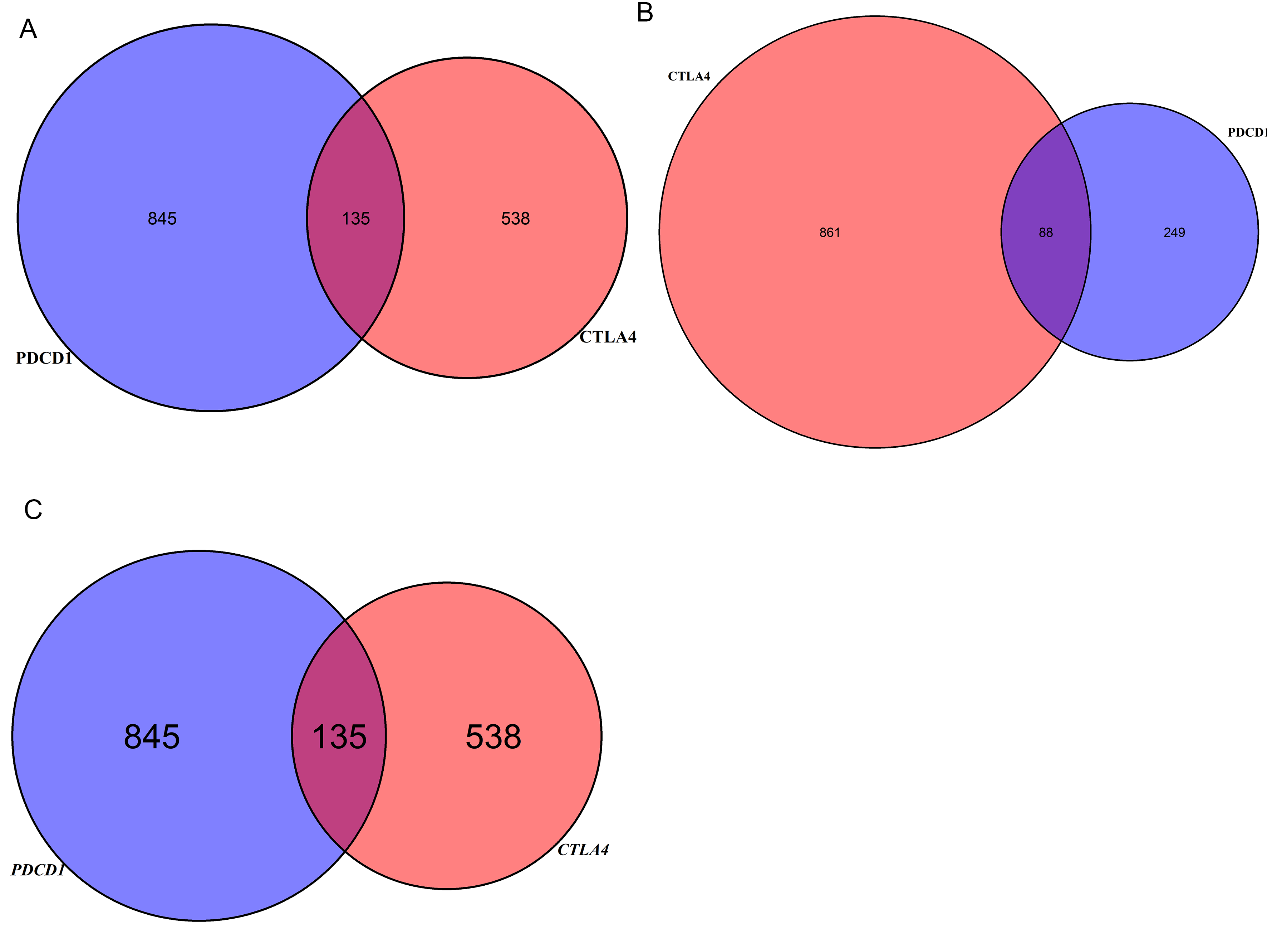


**Supplementary Figure 1.** Common dysregulated genes and biological processes in PDCD1 or CTLA4 cohort. A). Venn diagram of up-regulated genes. B). Venn diagram of down-regulated genes. C). Shared biological processes between PDCD1 and CTLA4 cohort.


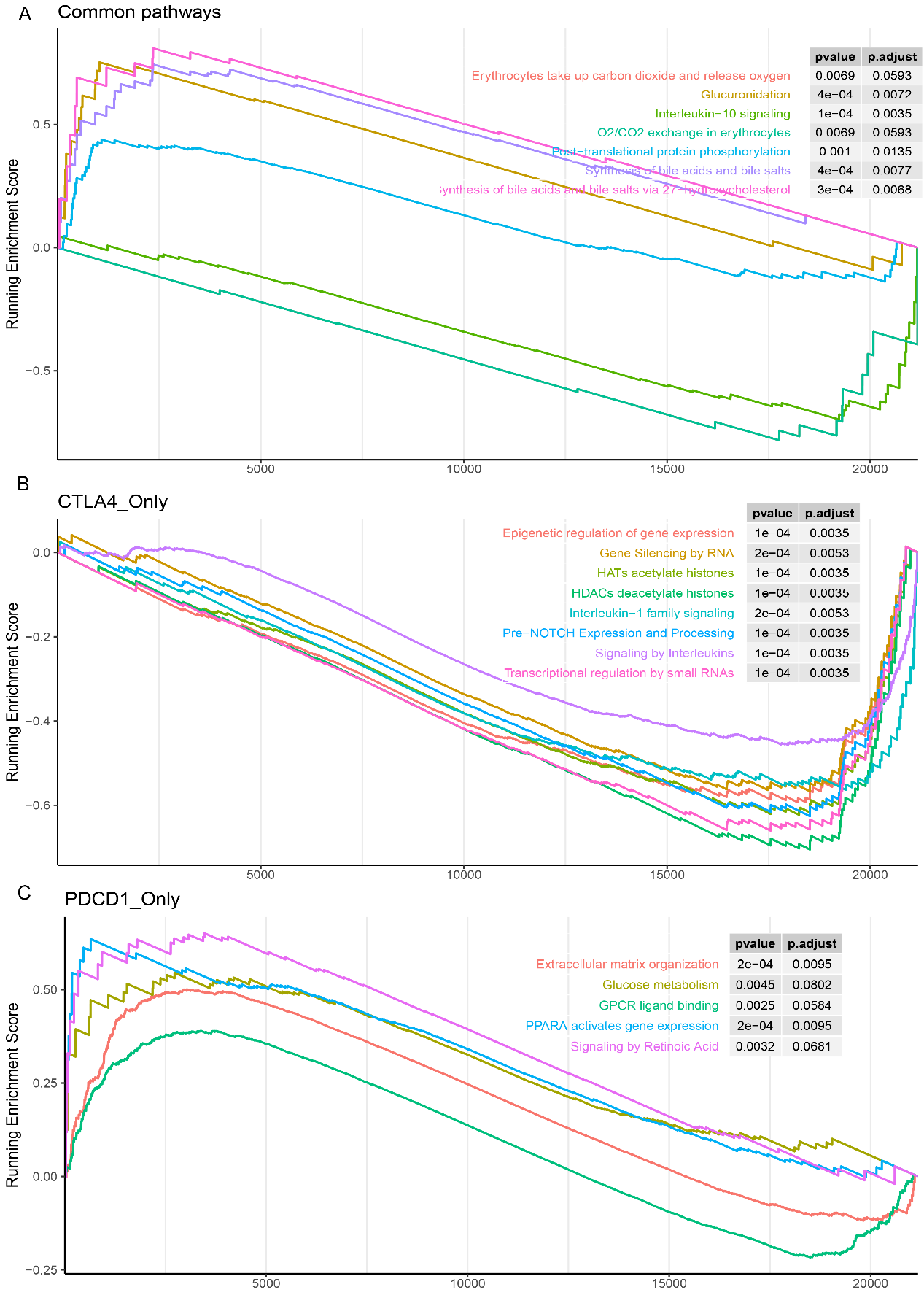


**Supplementary Figure 2.** Pathways enriched on PDCD1 and CTLA4 cohort. A). Shared pathways in PDCD1 and CTLA4 cohort. B). Pathways only enriched on CTLA4 cohort. C). Pathways only enriched on PDCD1 cohort.

**
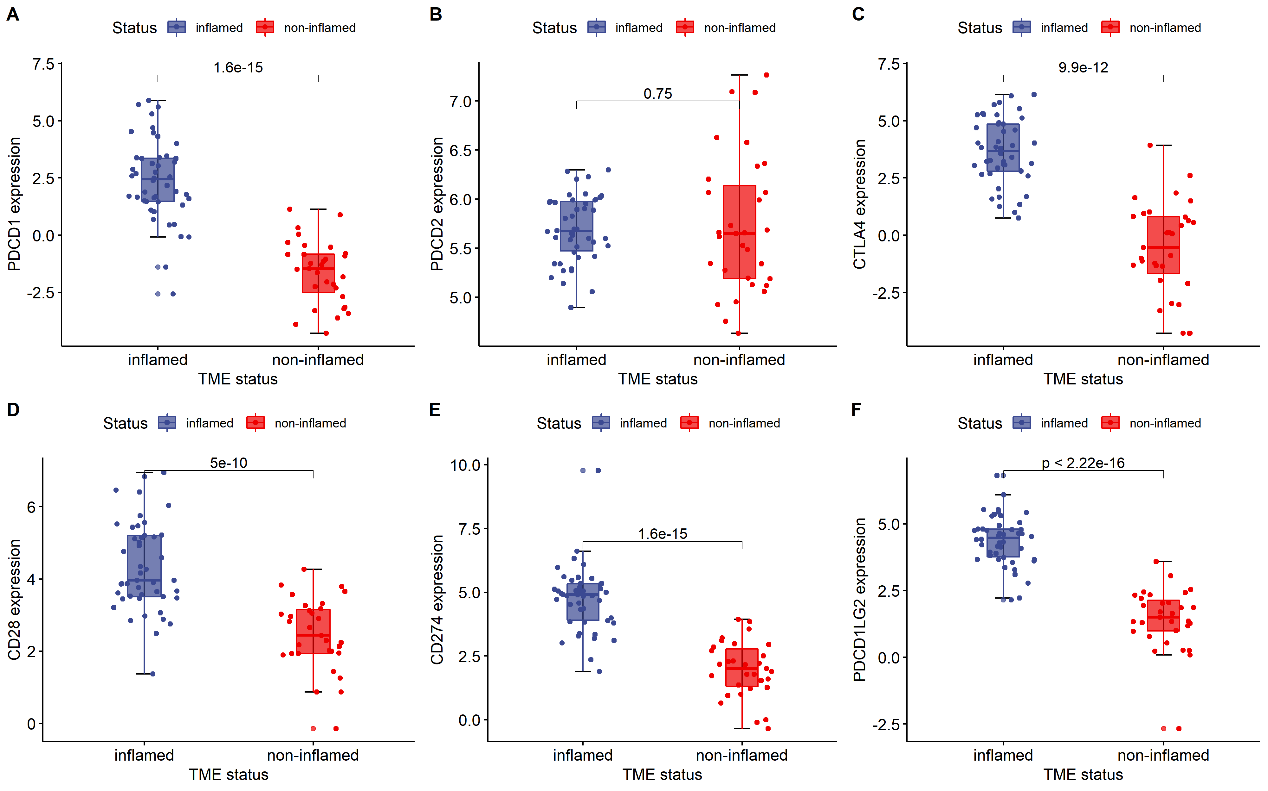
**

**Supplementary Figure 3.** The correlation between TME status with ICB response biomarker. A). PDCD1 B). PDCD2 C). CTLA4 D).CD28 E).CD274 F).PDCD1LG2


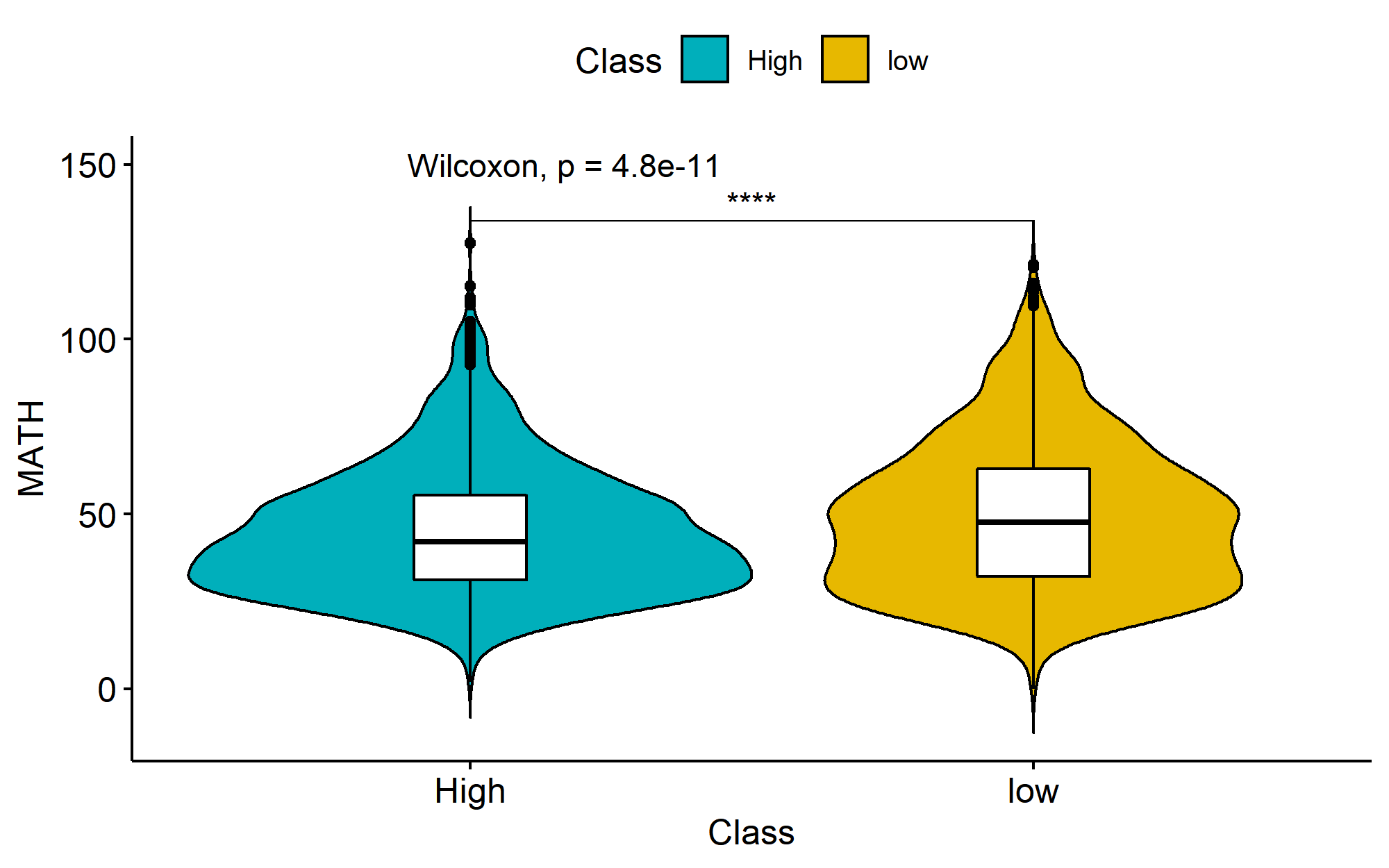


**Supplementary Figure 4.** The correlation between TME status with tumor mutation burden.
